# Service Differentiated and Adaptive CSMA/CA over IEEE 802.15.4 for Cyber-Physical Systems

**DOI:** 10.1155/2013/947808

**Published:** 2013-10-23

**Authors:** Feng Xia, Jie Li, Ruonan Hao, Xiangjie Kong, Ruixia Gao

**Affiliations:** School of Software, Dalian University of Technology, Dalian 116620, China

## Abstract

Cyber-Physical Systems (CPS) that collect, exchange, manage information, and coordinate actions are an integral part of the Smart Grid. In addition, Quality of Service (QoS) provisioning in CPS, especially in the wireless sensor/actuator networks, plays an essential role in Smart Grid applications. IEEE 802.15.4, which is one of the most widely used communication protocols in this area, still needs to be improved to meet multiple QoS requirements. This is because IEEE 802.15.4 slotted Carrier Sense Multiple Access/Collision Avoidance (CSMA/CA) employs static parameter configuration without supporting differentiated services and network self-adaptivity. To address this issue, this paper proposes a priority-based Service Differentiated and Adaptive CSMA/CA (SDA-CSMA/CA) algorithm to provide differentiated QoS for various Smart Grid applications as well as dynamically initialize backoff exponent according to traffic conditions. Simulation results demonstrate that the proposed SDA-CSMA/CA scheme significantly outperforms the IEEE 802.15.4 slotted CSMA/CA in terms of effective data rate, packet loss rate, and average delay.

## 1. Introduction

The new electrical power grid, which is also called as the Smart Grid, is among the high priority critical infrastructures of a nation [1]. It is an energy generation, transmission, and distribution system for improved efficiency, reliability, and safety, with smooth integration of renewable and alternative energy sources. Simultaneously, it provides a greater level of energy usage choice and automation that makes a grid truly “smart.” This is because the emerging Cyber-Physical Systems (CPS) in Smart Grid can enable the real-time monitoring of the current operational state of the network and respond to those conditions automatically, as soon as possible [2, 3]. Hence, CPS has been considered to be the next revolution in the field of information technology beyond computer and Internet [4], and its adoption for Smart Grid has aroused significant concern in the past several years.

In a Smart Grid system, CPS generally bridges the cyber and the physical worlds based on Wireless Sensor/Actuator Networks (WSANs) [1, 5], which serve as the network infrastructure of diverse CPS applications. WSANs play an essential role in this interactive process, since they are the medium of sensing and monitoring the physical world, especially considering that Smart Grid applications are highly dependent on CPS to accurately synchronize the real-world status on backend systems and processes and hence must sense and monitor the physical world safely, dependably, efficiently, and in real-time [6]. Therefore, new Quality of Service (QoS) requirements in the design of WSAN communication protocols have been put forward. The IEEE 802.15.4 [7], in particular, is a short-range, low-complexity, and low-power communication protocol for Low-rate Wireless Personal Area Networks (LR-WPANs). It can provide low-cost services and maximize the practical value of relevant achievements. However, IEEE 802.15.4 protocol needs to be optimized to meet the QoS requirements of Smart Grid CPS applications. This is because IEEE 802.15.4 Medium Access Control (MAC) protocol was not designed for networks that can provide QoS guarantees, while the performance of CPS applications are always dependent on the QoS of underlying networks. Therefore, it becomes important and necessary to improve the IEEE 802.15.4 MAC in the context of CPS in Smart Grid, which forms the focus of the paper.

In this paper, based on the IEEE 802.15.4 slotted (Carrier Sense Multiple Access/Collision Avoidance) CSMA/CA MAC protocol, we present an optimized algorithm: Service Differentiated and Adaptive CSMA/CA (SDA-CSMA/CA). To meet the QoS requirements of different data flows in CPS, this algorithm introduces the priority level to provide differentiated services to a number of devices. To solve the problem that backoff exponent *BE* cannot adapt to the current network status very well during transmission, an Adaptive Backoff Mechanism has also been proposed, in which the backoff time is dynamically set according to the current network status. Finally, the proposed SDA-CSMA/CA algorithm is implemented using the OMNeT++ simulator and an extensive evaluation of performance is given. Simulation results show that SDA-CSMA/CA is able to meet the QoS requirements of different data flows, with improved network reliability and utilization of network resources, which has a significant value in designing future MAC protocols for WSANs in Smart Grid CPS.

The remainder of this paper is organized as follows.  Section 2 gives an overview of related work in the literature. In Section 3, we introduce Smart Grid, CPS, the IEEE 802.15.4 standard, and their relevant features for subsequent discussions.  Section 4 proposes the SDA-CSMA/CA algorithm to provide differentiated and adaptive services for WSANs in Smart Grid CPS. Performance evaluation of SDA-CSMA/CA is presented in Section 5. Finally, Section 6 concludes the paper.

## 2. Related Work

Smart Grid has been a hot topic since it was first introduced in the paper “Toward a Smart Grid,” by Amin and Wollenberg in 2005 [8]. During the past several years, a large number of conferences, workshops, and summits on Smart Grid have been held, gathering researchers, practitioners, and leaders all around the world to discuss the challenges and opportunities brought by Smart Grid [9–11].

More recently, the CPS has emerged as a promising direction to enrich the interactions between physical and virtual worlds [12], and the adoption of which for Smart Grid is gaining wide attention from both the industry and the academia. Already today numerous research and deployment projects are under-way worldwide, investigating aspects of the Smart Grid including its CPS nature [6]. For example, according to ABI Research [3], an estimated $4.8 billion will be spent on the smart meters only for installing them at homes.

Most of the research that has been done on Smart Grid CPS is focused on the issue of energy consumption [13, 14]. However, reliability and timeliness guarantees have not been addressed in many cases, which may lead to unacceptable and serious results since CPS applications tend to be highly dependent on the QoS of underlying networks especially when they are used in Smart Grid. Recently, some researchers have been concerned about the QoS in WSANs and have developed some solutions [15–17]. For instance, Paek and Govindan [18] and Zhu et al. [19] studied real-time and dependable communication protocols. Firoze et al. [20] discussed MAC protocol with service differentiation. Slama et al. [21] and Yahya and Benothman [22] applied Hybrid MAC protocol to guarantee QoS. Nguyen et al. [23] designed a priority-based MAC to improve resource efficiency for multihop wireless networks.

Some studies have been conducted on service differentiation by configuring MAC parameters (e.g., *BE*, *CW*, *NB*) based on IEEE 802.15.4. For example, Kouba et al. [24] supported priority by allocating different *m* *a* *c* *M* *i* *n* *BE*s, *m* *a* *c* *M* *a* *x* *BE*s and *CW*s for different transmission classes. Ndih et al. [25] and Mohameden et al. [26] realized differentiated services by setting different *CW* and *BO* values. Kim and Kang [27] set different *CW*s or *m* *a* *c* *M* *i* *n* *BE*s to support service differentiation and compared the effectiveness of both. However, they did not consider the dynamic change of application requirements and network status, and hence their applicability in CPS needs to be further examined.

In this paper, based on our previous work [28, 29], we first examine the QoS requirements of CPS in Smart Grid and propose an optimization algorithm for IEEE 802.15.4 slotted CSMA/CA: SDA-CSMA/CA. This algorithm supports differentiated services for devices with different priorities and provides dynamic adaptation to network status (e.g., traffic conditions).

## 3. Background

In this section, we will briefly introduce Smart Grid, Cyber-Physical Systems, IEEE 802.15.4, and their relevant features for subsequent discussions in the paper.

### 3.1. Cyber-Physical Systems for Smart Grid

The term Smart Grid refers to a digitally enabled electrical grid that gathers, distributes, and acts on information about the behavior of all participants (suppliers and consumers) to maximize the throughput of the national electrical delivery system while reducing energy consumption. It enhances the electrical power grid to enable important advances in reliability, efficiency, economics, and sustainability of electricity services. Smart Grid builds on many existing technologies used by electric utilities, with additional communication and control capabilities to optimize the operation of the entire electrical grid. However, to meet the power quality and power availability demands of the 21st century, it is undergoing a major renovation. In order to provide the envisioned functionality of Smart Grid, we have to heavily depend on Cyber-Physical Systems (CPS) to monitor, share, and manage information and actions on the business as well as the real world. Hence, CPS is an integral part of Smart Grid, which provides the “glue” between the physical world and the business side [6].

**Figure 1:**
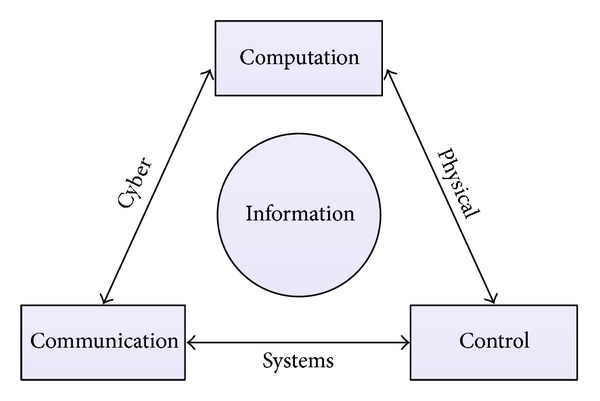
Cyber-Physical Systems.

CPS represents a coupling of computational and physical properties and can be found extensively in Smart Grid applications. The core idea behind this coupling is to seamlessly gather any useful information about objects of the physical world and use the information in various applications during the object's entire life cycle. In the design of the next generation intelligent buildings [30], for example, CPS which provides rapid access to information and decision-making can enable buildings to autonomously interact with the grid and allow buildings to participate in new real-time electricity markets. Such participation will be mutually beneficial reducing the energy costs of buildings and meanwhile, helping to manage peak loads, identify and reduce waste, and facilitate the integration of distributed renewable generation. CPS is arguably lying in the heart of the emerging Smart Grid. The abilities to interact with and expand the capabilities of the physical world through convergence of computation, communication, and control (Figure 1), which enables advanced monitoring and management of real-world processes, making it indispensable.

The basic components of a typical CPS include physical objects, sensors, actuators, communication networks, and computing devices. Various sensors and actuators will be geographically distributed and directly coupled with physical objects. Sensors are responsible for collecting the state information of physical objects and sending it to certain computing nodes through the communication networks, which could possibly be a combination of multiple networks of different types, for example, wired and wireless networks. Relatively complex decision making algorithms will be generally executed on computing devices. These devices are able to generate control commands based on information collected by sensors, which could be completed in a distributed or centralized manner. The control commands will then be sent to actuators and performed by them, also via networks if needed. In this way, CPS eases the integration of the physical and cyber worlds, that is, control of physical environments.

From the above we can see that CPS is generally built on WSANs. Through the networks within a real-world, CPS could potentially be much more complex and heterogeneous. In particular, when the scale of a CPS is very large, WSAN becomes a natural choice for interconnection of a large number of sensor, computing, and actuator nodes due to the celebrated benefits of wireless networking (as compared to wired counterparts). The use of WSAN distinguishes CPS from traditional embedded systems and wireless sensor networks. From a networking point of view, the Smart Grid CPS can be characterized as having the following features: resource constraints, platform heterogeneity, dynamic network topology, and mixed traffic.

### 3.2. QoS Requirements of Smart Grid CPS

The Smart Grid is regarded as the key part in a global ecosystem of interacting entities and behind which one of the major driving forces is the optimal management of available resources (locally and globally). To meet technical requirements for power that is reliable, efficient, economic, and environmentally responsible and to achieve the modern grid, a carefully designed CPS offering fine-grained monitoring and management is absolutely needed.

Unlike traditional embedded systems, a full-fledged CPS in Smart Grid will be able to improve the link between computational and physical elements, dramatically increasing the adaptability, autonomy, efficiency, functionality, reliability, safety, and usability. However, the integration of various subsystems, while keeping the system functional and operational, has been time-consuming and costly. Meanwhile, the increasing complexity of components and the use of more advanced technologies for sensors and actuators, wireless communication, and multicore processors also pose a major challenge for building next generation CPS [31]. Hence, how to make CPS both effective and efficient in a complex system of systems such as the Smart Grid has become a crucial issue in the research field of future energy systems.

The Smart Grid CPS will ultimately require hundreds of standards, specifications, and requirements, some of which are urgently needed, as is addressed by NIST in the report on Smart Grid interoperability standards roadmap [32]. 

*Network Communications*. In the various networking environments of the Smart Grid system, the identification of performance metrics and operational requirements of different applications, actors, and domains is crucial to the Smart Grid. This includes the Quality of Service (QoS) support for a wide range of applications with different latency and loss requirements as well as the specific relative priorities for individual Smart Grid applications. 

*Traffic Management*. In order to increase reliability, reduce peak loads, and improve capabilities for controlling distributed energy sources, we need to focus on maximizing performance of networked distribution and transmission systems. The number of devices, the amount of data, and communication frequencies are necessary to be carefully determined. Traffic conditions should also be considered for every type of data transmissions.

Some widely recognized QoS parameters can be outlined as follows [33, 34].

*( 1) Average Delay.* It is a crucial metric to evaluate the real-time performance of networks, which refers to the average time experienced by a data packet from the start of its generation to the end of its reception. 

*( 2) Effective Data Rate.* It is used to evaluate the link bandwidth utilization which reflects the resource efficiency as well as dependability of networks. 

*( 3) Packet Loss Rate.* It is computed as the traffic dropped by the network divided by the overall traffic, which clearly reflects the degree of reliability achieved by CPS for successful transmissions.

Based on the above analysis, in this paper we focus our attention on the network (e.g., IEEE 802.15.4) QoS in CPS in terms of these properties.

### 3.3. Relevant Features of IEEE 802.15.4 MAC

The IEEE 802.15.4 standard [7] is designed for low-rate, low-power, and low-cost Personal Area Networks (PANs). A typical IEEE 802.15.4 network mainly consists of the following components: the PAN coordinator, which manages the entire network; one or more of coordinators, which manage a cluster of nodes; and multiple ordinary nodes that are associated with certain coordinator to participate in network operations.

As for the channel access, IEEE 802.15.4 MAC protocol supports two operational modes that may be selected by the PAN coordinator: the beacon enabled mode and the nonbeacon enabled mode. In the beacon mode, the PAN coordinator transfers the beacon frames periodically to all nodes within its radio coverage. All the nodes within the radio coverage are synchronized by these beacon frames. The nonbeacon mode only supports contention-based access through unslotted CSMA/CA with no use of the beacons and yields longer delay and more energy consumption. Hence, we select the beacon enabled mode as a basic operating mode in consideration. 

**Figure 2:**
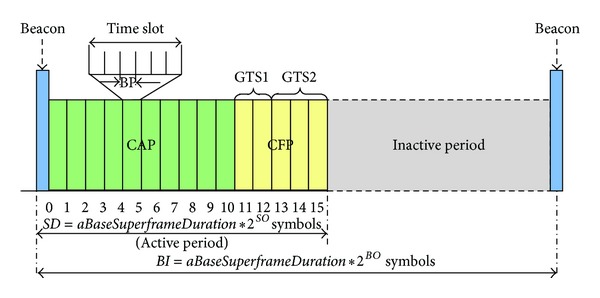
Superframe structure of IEEE 802.15.4.

A superframe structure (Figure 2) is used in the beacon enabled mode, which the PAN coordinator uses to synchronize nodes that are associated with it. The superframe structure is further characterized by a *Beacon* *Interval*  (*BI*) specifying the time between two consecutive beacons, and a *Superframe* *Duration* (*SD*) corresponding to the active period, defined as shown below:

(1) BI=aBaseSuperframeDuration∗2BO,for  0≤BO≤14,SD=aBaseSuperframeDuration∗2SO,for  0≤SO≤BO≤14,

where *BO* and *SO* are called *Beacon* *Order* and *Superframe* *Order*, respectively. The *BI* may optionally include an inactive period (for *SO* < *BO*), in which all nodes may enter into a sleep mode, thus, saving energy. More details can be found in [7].

**Figure 3:**
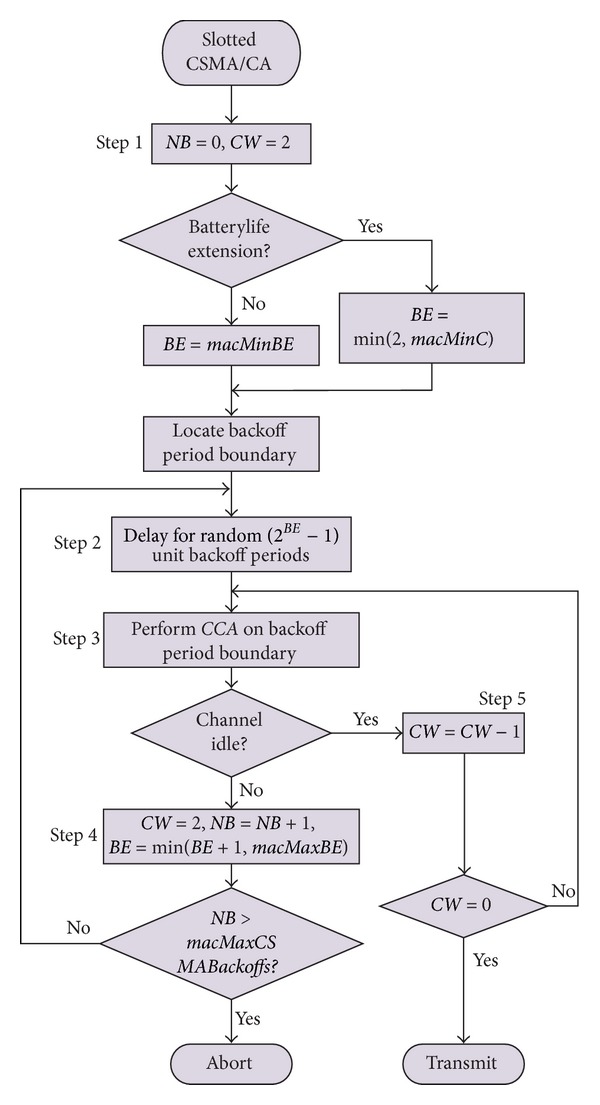
The slotted CSMA/CA mechanism.

The beacon-enabled IEEE 802.15.4 MAC also supports a slotted CSMA/CA mechanism based on a basic time unit called *Backoff* *Period*  (*BP*), which is equal to *a* *U* *n* *i* *t* *Backoff* *Period* = 80 bits  (0.32 ms). This CSMA/CA operation is controlled by three parameters: contention window (*CW*), backoff exponent (*BE*), and number of backoff (*NB*). The flowchart shown in Figure 3 describes the slotted CSMA/CA mechanism in more detail.

The algorithm begins by setting *NB* to 0 and *CW* to 2 (Step 1). If the device operates on battery power, *BE* is set to 2 or to the constant *m* *a* *c* *M* *i* *n* *BE*, whichever is less; otherwise, it is set to *m* *a* *c* *M* *i* *n* *BE* (the default value of which is 3). The algorithm then locates the boundary of the next backoff period. Then, the algorithm starts counting down a random number of *BP*s uniformly generated within [0, 2^*BE*^ − 1] (Step 2). The countdown must start at the boundary of a *BP*. When the timer expires, the algorithm then performs one *CCA* operation at the *BP* boundary to assess channel activity (Step 3). If the channel is busy, the values of *NB* and *BE* are increased by one (but *BE* cannot exceed *m* *a* *c* *M* *a* *x* *BE*, the default value of which is 5), while *CW* is reset to 2 (Step 4). If the number of retries is below or equal to *m* *a* *c* *M* *a* *x* *C* *S* *M* *A* *Backoff* *s* (the default value of which is 5), the algorithm returns to Step 2; otherwise, the algorithm terminates with a channel access failure status. If the channel is idle (Step 5), the value of *CW* is decreased by one, and the channel is assessed again. Packet transmission may begin when *CW* reaches 0.

### 3.4. Analysis of IEEE 802.15.4 Slotted CSMA/CA MAC

Due to the above-described QoS requirements of Smart Grid CPS and characteristics of IEEE 802.15.4 MAC, we can easily observe that the slotted CSMA/CA MAC specified in the IEEE 802.15.4 standard may not be suitable for CPS in Smart Grid.

Service differentiation, as an essential component of QoS, should be supported by communication protocols. CPS may encompass diverse applications in a Smart Grid system, which may differ significantly in their QoS requirements. For example, in applications like audio and video data streams, Advanced Metering Infrastructure (AMI) periodic measurements, and so forth, data should be transmitted immediately when generated, while data like medium speed monitoring and control information should be given a comparatively lower priority and a longer delay may be acceptable. As a result, communication protocols for WSANs in CPS should be designed to perceive the service requirements of each type of traffic so that it can be guaranteed a specific service level. However, the best-effort service offered by original IEEE 802.15.4 slotted CSMA/CA MAC cannot provide different QoS to different applications, where all devices compete for the medium access using contention-based CSMA/CA during the CAP. In practice, the best-effort service is likely to be the standard for the foreseeable future. It is therefore necessary for new QoS mechanisms to be layered on top of the existing networks.

Furthermore, adaptability is of paramount importance for QoS provisioning, because of the ever-increasing complexity of CPS in Smart Grid, highly dynamic feature of the networks, and changing and unpredictable environments. However, the legacy IEEE 802.15.4 can hardly provide adaptation to multiple QoS requirements of CPS due to its static parameter setting policy. For instance, the IEEE 802.15.4 states that the *BE* value is set to a random number between the range of two variables, *m* *a* *c* *M* *i* *n* *BE* and *m* *a* *c* *M* *a* *x* *BE*. This is without consideration of traffic conditions. A certain value of *BE* may be suitable for light traffic environments, while leading to severe collisions when there is a heavy traffic. Similarly, an appropriate *BE* in heavy traffic conditions will probably results in plenty of time wasted before a *CCA* operation in a light traffic environment. In this way, the traditional inflexible IEEE 802.15.4 slotted CSMA/CA MAC will definitely decrease the overall efficiency of Smart Grid CPS and significant efforts will be needed to achieve the modern grid with envisioned functionalities.

## 4. SDA-CSMA/CA: Service Differentiated and Adaptive CSMA/CA

The objective of this section is to propose the SDA-CSMA/CA algorithm for IEEE 802.15.4 standard to solve the problems raised in Section 3.4. Our SDA-CSMA/CA adopts a priority-based dynamic parameter setting approach, which includes a Service Differentiation Mechanism and an Adaptive Backoff Mechanism. Both mechanisms cooperate to minimize delay of emergent packets and improve overall QoS performance in terms of effective data rate, packet loss rate, number of collisions, and so forth.

### 4.1. Service Differentiation Mechanism

It is widely known that data packets may have various levels of importance in Smart Grid CPS. There is often a clear distinction between important and less-important data packets that can be found in a variety of Smart Grid applications. For instance, the data packets containing critical information, such as the system protection and lockout functions, are more important than the data packets for distribution applications and monitoring and control information. Hence, service differentiation is needed to enable devices that produce emergency data to take priority over other devices.

In service differentiation or prioritization, we can use two types of access parameters: *CW* and *BE*. *CW* represents the number of *CCA*s, which is performed prior to each packet transmission to determine whether the medium is busy or idle. The IEEE 802.15.4 standard specifies that the transmitter node performs the *CCA* twice in order for the purpose of protecting acknowledgement frame and giving enough time for a receiving device to process the frame. However, it is easy to see that a bigger *CW* (i.e., 3) will give a lower priority to obtain the channel, compared with the default 2, thus, differentiating services with various levels of importance.

*BE* is related to the number of *BP*s that a device must wait before attempting to access the channel. The *m* *a* *c* *M* *i* *n* *BE* indicates the minimum number of *BE*, whose default value is given as 3. And *m* *a* *c* *M* *a* *x* *BE* indicates the maximum number of *BE*, which is limited to 5 in the standard. Yet, to give differentiation to individual nodes, we suggest that both *m* *a* *c* *M* *i* *n* *BE* and *m* *a* *c* *M* *a* *x* *BE* are flexible to changes. A smaller *m* *a* *c* *M* *i* *n* *BE* or *m* *a* *c* *M* *a* *x* *BE* will shorten the waiting time when *CCA* detects the channel busy or when a packet collides with a different packet in the channel. This gives a higher possibility of making a successful transmission and throughput increment compared to other nodes with longer waiting times.

Therefore, our proposed Service Differentiation Mechanism is based on varying the parameters, *CW*s, *m* *a* *c* *M* *i* *n* *BE*s, and *m* *a* *c* *M* *a* *x* *BE*s according to the priority of a device. For this purpose, we classify all devices into three levels of importance: *L*1 > *L*2 > *L*3 and parameter settings of each priority level are different. Smaller *CW*s, *m* *a* *c* *M* *i* *n* *BE*s, and *m* *a* *c* *M* *a* *x* *BE*s are assigned to high-priority sensor nodes, respectively, in comparison with low-priority ones. This allows important messages to have more chance to access the channel with shorter backoff time. However, the *CW* and *m* *a* *c* *M* *i* *n* *BE* values can neither be too large nor too small. This is because extremely large *CW*s and *m* *a* *c* *M* *i* *n* *BE*s will lead to unnecessary delays in data transmissions and extremely small values may cause severe collisions in network, especially among high-priority devices. To avoid these phenomenon, we have the same *CW* (*CW* = 2) for priority level *L*1 and *L*2 and differentiate them via setting different *m* *a* *c* *M* *i* *n* *BE* values. Similarly, *L*2 and *L*3 have the same *m* *a* *c* *M* *i* *n* *BE* (*m* *a* *c* *M* *i* *n* *BE* = 3) and they can be differentiated by different *CW*s. Note that in this case, we do not have much restriction on *m* *a* *c* *M* *a* *x* *BE* settings since it is hardly accessed in normal network conditions, where there is always only a proper amount of collisions. Hence, we set *m* *a* *c* *M* *a* *x* *BE* values to priority levels in a more relaxed manner. In this way, by varying parameters, *CW*s, *m* *a* *c* *M* *i* *n* *BE*s, and *m* *a* *c* *M* *a* *x* *BE*s, devices are prioritized and differentiated services can be rendered. The parameter settings for each of the three levels are shown, respectively, in Table 1.

### 4.2. Adaptive Backoff Mechanism

*BE* is the number of Backoff Periods before the *CCA* operation. The larger the *BE*, the longer the waiting time for a device before it attempts to access the channel. Hence, the value of *BE* has a significant impact on network efficiency.

The proposed Adaptive Backoff Mechanism is aimed to assign dynamic and adaptive *BE*s to data flows in consideration of different traffic conditions. This provides a good estimate of future traffic load in the IEEE 802.15.4-based WSANs; thus, *BE* values can properly be adjusted to different circumstances.

Specifically, we assume that each device maintains the following parameters at the beginning of the *t*th superframe: *P*_0_^*t*^, *P* ^*t*−1^, and *P* ^*t*^. *P*_0_^*t*^ is a clear reflection of current network traffic load. It can be computed as (*NB*)^*t*^ divided by *N*_*CCA*_^*t*^, where (*NB*)^*t*^ represents the number of backoffs in the *t*th superframe and *N*_*CCA*_^*t*^ is the overall number of *CCA*s operated in the *t*th superframe. According to the CSMA/CA algorithm described in Section 3, we can observe that for the same *CCA*s, a larger *NB* usually indicates a much heavier traffic load in network compared with a smaller *NB*. Hence, we can get a good understanding of network traffic conditions from the ratio of *NB* to *N*_*CCA*_, and based on this information, *BE* can be dynamically adapted to its particular network environment in our proposed algorithm. The equation of *P*_0_^*t*^'s definition is shown as follows:

(2) $$\begin{array}{c} P_{0}^{t}=\frac{{\left(NB\right)}^{t}}{N_{CCA}^{t}}. \end{array}$$

Further, in order to effectively minimize the jitter generated in the process, a sliding mean average value *P* ^*t*^ is calculated as shown in the following equation below:

(3) $$\begin{array}{c} P^{t}=\alpha \times P_{0}^{t}+\left(1-\alpha \right)\times P^{t-1}, \end{array}$$

where *P* ^*t*^ and *P* ^*t*−1^ indicate traffic load in the current (*t*th) superframe and the previous ((*t* − 1)th) superframe, respectively. *P* ^*t*^ is initialized to 0 at the very beginning. *α* is a constant and 0 ≤ *α* ≤ 1.

Furthermore, given the previously described priority levels, we define *P*_min⁡_[*level*] and *P*_max⁡_[*level*] as the thresholds of traffic load *P* ^*t*^ for devices in different levels. Then *BE*s can be dynamically set as shown in the following process (Algorithm 1), where *BE*0 represents the the value of *BE* after the completion of the previous SDA-CSMA/CA and before the current SDA-CSMA/CA starts. It is the initial *BE* value in the dynamic setting process.

**Figure 4:**
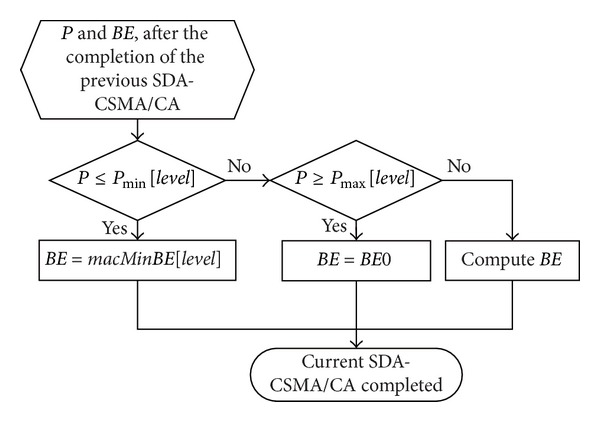
*BE* dynamic initialization.

Figure 4 depicts the *BE* dynamic initialization process in the flowchart.

Services are further differentiated through varying *P*_min⁡_[*level*] and *P*_max⁡_[*level*] according to their different levels of importance. In this process, we can find that a larger *P*_min⁡_[*level*] gives a higher chance of obtaining a small *BE*, whereas a smaller *P*_min⁡_[*level*] usually indicates a lower probability of obtaining a small *BE*. Similarly, for *P*_max⁡_[*level*], its setting also has a significant impact on the *BE* values. Hence, in order to shorten the waiting time of high-priority devices, both large *P*_min⁡_[*level*] and *P*_max⁡_[*level*] values should be assigned. In contrast, lower priority level devices should be given smaller *P*_min⁡_[*level*] and *P*_max⁡_[*level*] values in comparison. Hence, based on the above discussions, we give the *P*_min⁡_[*level*] and *P*_max⁡_[*level*] settings for each priority level in Table 1. 

**Figure 5:**
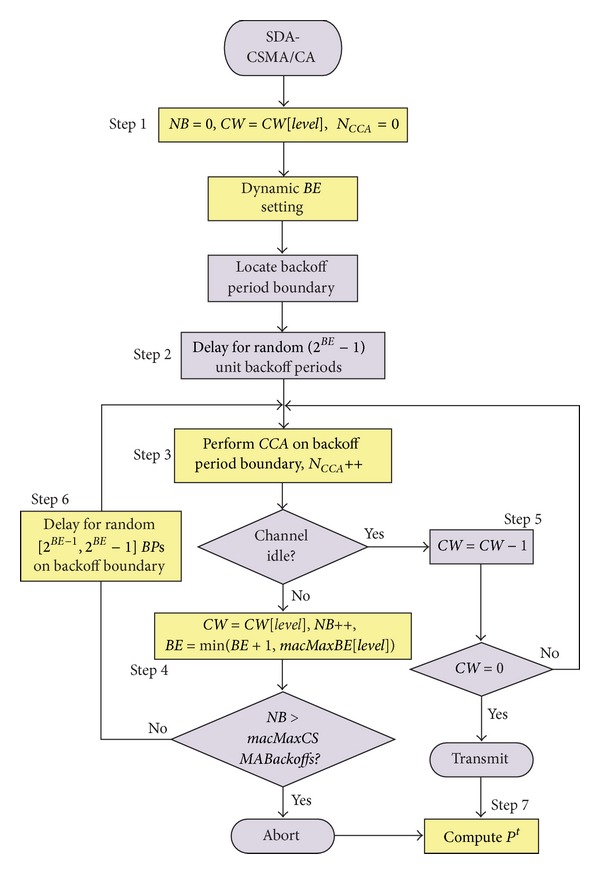
SDA-CSMA/CA algorithm.

In summary, Figure 5 presents the flowchart of the SDA-CSMA/CA algorithm. Upon receiving a data frame to be transmitted, the SDA-CSMA/CA algorithm performs the following steps: (Note that steps with a yellow background are the places where the original IEEE 802.15.4 slotted CSMA/CA MAC is improved.)

1. A set of state variables are initialized: the contention window size (*CW* = *CW*[*level*]), the number of backoff stages carried out for ongoing transmissions (*NB* = 0), and the backoff exponent (adaptively adjusted to current traffic load as described in the Dynamic *BE* Setting process).
2. A random backoff time, uniformly distributed in the range [0,320 × (2^*BE*^ − 1)] *μ*s is generated to initialize a backoff timer.
3. A *CCA* is performed to check the state of the wireless medium and *N*_*CCA*_ is increased by one.
4. If the medium is busy, the state variables will be updated: *NB* = *NB* + 1, *BE* = min⁡(*BE* + 1, *m* *a* *c* *M* *a* *x* *BE*[*level*]), and *CW* = *CW*[*level*]. If *NB* exceeds the maximum admissible value (*m* *a* *c* *M* *a* *x* *C* *S* *M* *A* *Backoff* *s*), the frame is dropped. Otherwise, Step 6 will be performed.
5. If the channel is accessed to be idle, the MAC sublayer must ensure that the contention window is expired before starting transmission. For this, the MAC sublayer first decrement *CW* by one. If *CW* is not equal to 0, it must go back to Step 3 to the Clear Channel Assessment Phase. If the channel is again sensed as idle, the MAC shall start transmission immediately.
6. A random number is selected in the range 2^*BE*−1^ to 2^*BE*^ − 1 to set the backoff timer.
7. *P* ^*t*^ is computed in this step for *BE*'s initialization when next time SDA-CSMA/CA is performed.

## 5. Performance Evaluation

In this section, we conduct a simulation study based on an accurate model of IEEE 802.15.4 in OMNeT++ and access the QoS performance of our proposed SDA-CSMA/CA algorithm, compared with the original IEEE 802.15.4 slotted CSMA/CA MAC for Smart Grid CPS.

### 5.1. Simulation Setup

**Figure 6:**
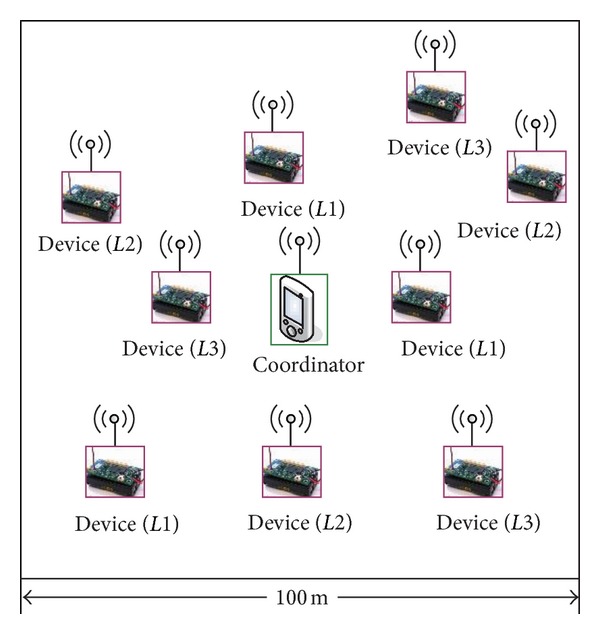
Simulated network topology.

Simulations are conducted using the IEEE 802.15.4 module included in the OMNeT++ simulator. In the simulation, a star topology with single PAN coordinator and a number of devices deployed in the area of 100 m × 100 m is considered (Figure 6). Each device transmits data packets to the coordinator and the transmission range of every node is 176 m so that we can easily learn that all the nodes are set to be in each other's radio range. Hence, there is no hidden node. All devices periodically generate a data packet addressed to the coordinator. In the Physical Layer, the 2.4 GHz range and a bandwidth of 250 kbps are used in simulations. In addition, some important fixed parameters and default values of variable parameters are listed in Table 2.

As mentioned in Section 3.2, the network QoS in CPS requires communication protocols to be both effective and efficient. To satisfy these needs, we use the three previously described QoS metrics, that is, Average Delay, Effective Data Rate, and Packet Loss Rate, to evaluate the proposed SDA-CSMA/CA algorithm in the paper. They are defined as follows. 

*( 1) Average delay*

(4) $$\begin{array}{c} D_{\text{ave}}=\frac{N_{\text{delay}}}{N_{\text{susspacket}}}, \end{array}$$

where *N*_delay_ is the total delay experienced by all data packets, that is, from the start of their generation to the end of their reception, and *N*_susspacket_ is the total number of data packets that are successfully received by the coordinator from all devices. 

*( 2) Effective data rate*

(5) $$\begin{array}{c} R_{\text{effData}}=\frac{N_{\text{susspacket}}\times L_{\text{MSDU}}}{T_{\text{end}}-T_{\text{start}}}, \end{array}$$

where *L*_MSDU_ is the MSDU length of the data frame. And the quantity *T*_end_ − *T*_start_ indicates the total time of transmission. 

*( 3) Packet loss rate*

(6) $$\begin{array}{c} R_{\text{loss}}=\frac{N_{\text{dropped}}}{N_{\text{generated}}}, \end{array}$$

where *N*_dropped_ represents the traffic dropped by the network while *N*_generated_ refers to the overall traffic. 

### 5.2. Numerical Results and Analysis

**Figure 7:**
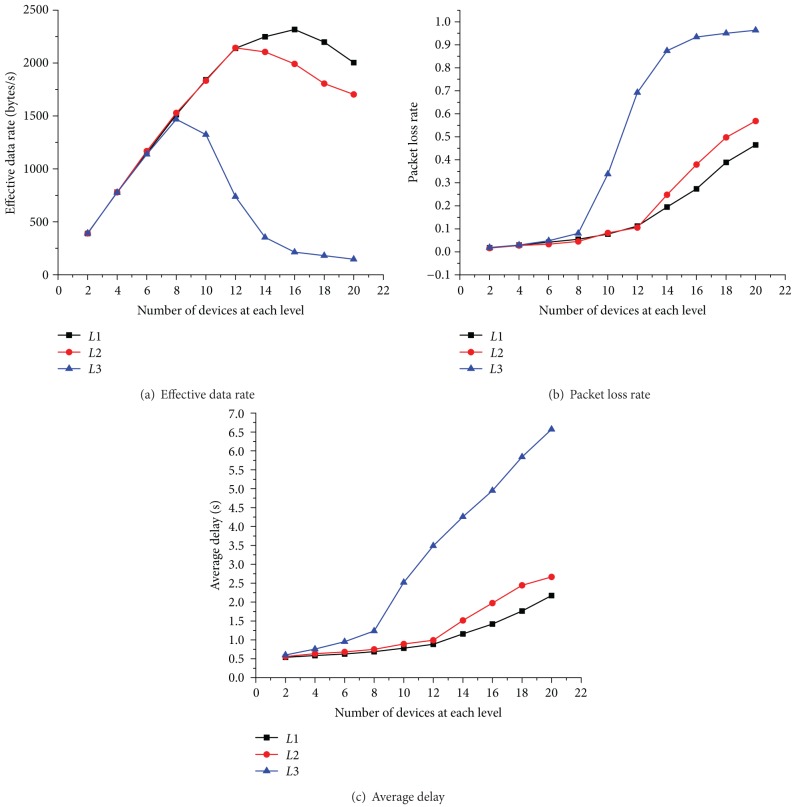
Performance comparison of different levels.

In this section, we present the simulation results and analysis of the proposed SDA-CSMA/CA algorithm in terms of average delay, effective data rate, and packet loss rate with multiple priority levels. In addition, similar assessments of the original IEEE 802.15.4 slotted CSMA/CA MAC have also been rendered for comparison. Hence, the detailed performance evaluation is presented in two parts: (1) performance comparison of Smart Grid applications in different priority levels (Figure 7) and (2) performance comparison of SDA-CSMA/CA and IEEE 802.15.4 slotted CSMA/CA MAC (Figure 7).

Figure 7(a) depicts the measured effective data rate, which first grows and then decreases with an increasing number of devices. The reason for this phenomenon is that as the number of devices increases, more packets are sent in the same time and traffic load increase. However, overly heavy traffic load may lead to a higher probability of collision, thus causing the decrease of effective data rate.

Figure 7(b) shows the measured packet loss rate, which increases with the number of devices in this context. One reason may be that in denser networks, more devices compete to access the channel at the same time, and consequently more packet collisions will happen.

Figure 7(c) presents the measured average delay. We can see that the curve trend in this figure is similar to that in Figure 7(b). From the above analysis of packet loss rate, we know that the increasing number of devices leads to the increasing probability of packet collisions. This can significantly contribute to the increase of times of backoff and retransmission and eventually lead to a longer average delay.

Furthermore, we can find that in Figures 7(a), 7(b), and 7(c), devices in higher priority levels tend to have higher effective data rate, lower packet loss rate, and lower average delay compared to those in lower levels. This is because of the Service Differentiation Mechanism in SDA-CSMA/CA. As we presented in Section 4.1, devices at each level have different parameter settings, including *CW*, *m* *a* *c* *M* *i* *n* *BE*, *m* *a* *c* *M* *a* *x* *BE*, *P*_min⁡_, and *P*_max⁡_, impact of which have already been discussed previously. These settings work together to provide QoS guarantee for high-priority devices and enable them to take priority over other devices in data transmissions.

**Figure 8:**
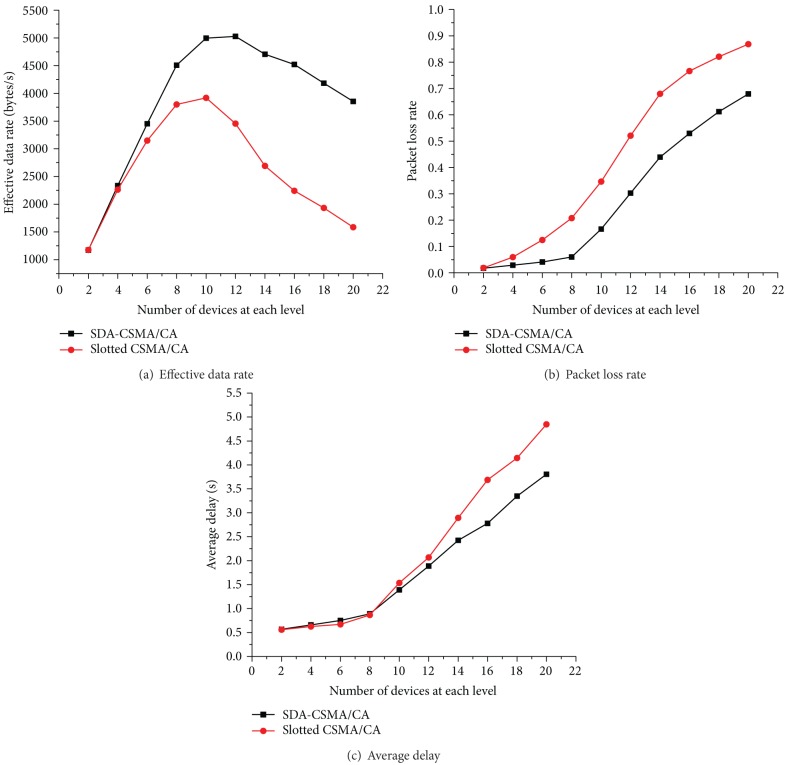
Performance comparison of SDA-CSMA/CA and IEEE 802.15.4 slotted CSMA/CA.

In order to have an overall understanding of the proposed SDA-CSMA/CA and original slotted CSMA/CA in comparison, we again use the three metrics to evaluate both the two schemes in simulation. Figures 8(a), 8(b), and 8(c) present the impact of the number of devices on effective data rate, packet loss rate, and average delay, respectively, where the (red) line with circle markers indicates the original slotted CSMA/CA specified in the IEEE 802.15.4 standard and the (black) line with square markers represents the proposed SDA-CSMA/CA algorithm.

Figure 8(a) shows the measured effective data rate, from which we can see that SDA-CSMA/CA has a better performance than the slotted CSMA/CA. This may results from the Adaptive Backoff Mechanism, which can adaptively adjust *BE* to traffic conditions. Specifically, in SDA-CSMA/CA, a smaller *BE* is used in backoff when there is a light traffic while a larger *BE* is assigned in heavy traffic environments. In this case, data packets are transmitted in a more effective and efficient manner, with less waiting time and fewer collisions. This leads to the higher effective data rate in SDA-CSMA/CA as shown in Figure 8(a).

Figure 8(b) shows the proposed scheme also has a lower packet loss rate than the IEEE 802.15.4 slotted CSMA/CA. This is because the slotted CSMA/CA is much more sensitive to traffic conditions. The dynamic parameter settings in SDA-CSMA/CA rather than the static setting specified in slotted CSMA/CA gives a strong guarantee for successful transmissions, especially when there is a heavy traffic. This will definitely results in fewer packet collisions and a lower possibility of packet loss in SDA-CSMA/CA.

Meanwhile, the Adaptive Backoff Mechanism in SDA-CSMA/CA also contributes to a lower average delay (Figure 8(c)), since dynamic *BE* settings give a shorter waiting time in light traffic conditions as well as mitigation of the heavy traffic to avoid packet collisions. Hence, less time is needed by a data packet from the start of its generation to the end of its reception.

## 6. Conclusions

In this paper, we have proposed a Service Differentiated and Adaptive CSMA/CA (SDA-CSMA/CA) algorithm based on a two-stage approach. In the first stage of the SDA-CSMA/CA, we employ a priority-based Serviced Differentiation Mechanism, in which different MAC parameters are set to diverse Smart Grid applications. The second stage of the proposed algorithm can dynamically adjust the backoff exponent to current traffic conditions, which is learnt from previous backoff behaviors. This can significantly contribute to both effective and efficient data transmissions for IEEE 802.15.4-based WSANs in Smart Grid CPS. The SDA-CSMA/CA algorithm is developed based on the IEEE 802.15.4 MAC protocol and fully compatible with the implementation of IEEE 802.15.4 devices. Numerous simulations show that SDA-CSMA/CA dramatically improves performance of CPS in Smart Grid, in terms of effective data rate, packet loss rate, and average delay compared to original slotted CSMA/CA MAC.

## Figures and Tables

**Algorithm 1:**
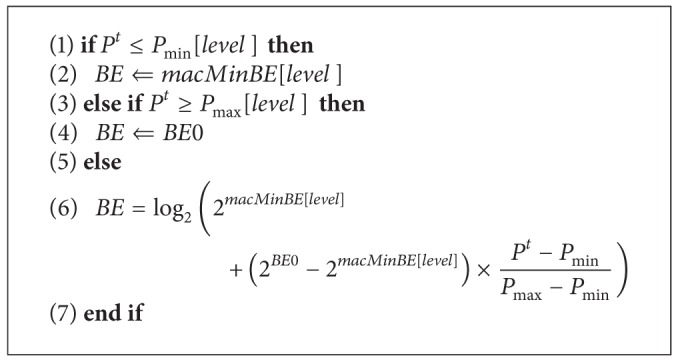
Dynamic *BE* setting.

**Table 1:** Parameter setting for each priority level.

Level	*CW*[*level*]	*ma* *cM* *in* *BE*[*level*]	*ma* *cM* *ax* *BE*[*level*]	*P*_min⁡_[*level*]	*P*_max⁡_[*level*]
*L*1	2	2	5	0.4	0.8
*L*2	2	3	5	0.3	0.7
*L*3	3	3	6	0.2	0.6

**Table 2:** Parameter settings.

Parameter	Value
Carrier frequency	2.4 GHz
Transmitter power	1 mW
Carrier sense sensitivity	−85 dBm
Transmission range	176 m
Bit rate	250 kbps
Traffic type	Exponential
Packet generation interval	0.25 s
Run time	1 h
Maxframeretries	3 (default)
MAC payload size (MSDU size)	50 bytes (default)
Superframe order (*SO*)	8 (default)
Beacon order (*BO*)	7 (default)
*α*	0.5
